# Congenital lumbosacral junction kyphosis in an adult patient: A case report

**DOI:** 10.1002/ccr3.8094

**Published:** 2023-10-23

**Authors:** Arvin Eslami, Mohammadreza Chehrassan, Shaya Alimoghadam, Mohammadreza Shakeri

**Affiliations:** ^1^ Bone and Joint Reconstruction Research Center, Shafa Yahyaeian Orthopedic Hospital Iran University of Medical Sciences Tehran Iran; ^2^ Department of Orthopedic, School of Medicine Iran University of Medical Sciences Tehran Iran; ^3^ School of Medicine Shahid Beheshti University of Medical Sciences Tehran Iran

**Keywords:** congenital anomalies, gait problem, hemivertebra, lordosis, lumbar, MRI spine, neurologic bladder, PSFI, sacral, urinary retention

## Abstract

Rare lumbosacral junction kyphosis due to S1–S2 hemivertebra in a 40‐year‐old woman was managed surgically, improving neurological disturbances, and low back pain. Early intervention is vital for congenital anomalies.

## 1 BACKGROUND

Hemivertebra (HV) is a rare congenital anomaly characterized by the complete failure of vertebral body formation.^1^ Among HV cases, sacral HV is exceptionally uncommon, with only seven reported cases prior to this (Table 1).^2^, ^3^, ^4^, ^5^, ^6^, ^7^ Posterior HVs are wedge‐shaped deformities caused by the failure of anterior vertebral formation, leading to progressive kyphosis.^8^, ^9^

**TABLE 1:** Previous case reports of sacral hemivertebra and associated symptoms in reported patients.

Author	Age	Level of sacral hemivertebra	Accompanying symptoms
Faivre et al.^2^	Child	Not mentioned	VACTERL
Darouich et al.^3^	Fetus	Not mentioned	Bilateral severe hypoplasia of the femora, hypoplastic and vertical pelvic bones
Dentici et al.^4^	6 years old	Not mentioned	Cleft palate, atrial septal defect (ASD), bicuspid aortic valve (BAV), Anal atresia, partial synostosis of C3–C4, C6–C7, T3–T4, lumbar scoliosis, unilateral postaxial polydactyly of the right hand
Fayard et al.^5^	Fetus	S3–S4	Bilateral hydronephrosis, two pelvic cystic masses behind the bladder, an abnormal rectum
Daher et al.^6^	10 months old	S1–S2	Anorectal malformation
Karaeminogullari et al.^7^	11 months old	S2–S3	Congenital heart disease

Lumbosacral HV typically does not pose a risk of kyphosis deformity, and no cases of kyphosis associated with lumbosacral HV were identified before this report.^10^ A sacral HV between S1 and S2 is an extremely rare condition, with only one previously reported case in a 10‐month‐old infant. However, there was no explanation provided about its clinical symptoms in adulthood or the potential problems caused by it.^11^

In this article, our aim is to describe the condition of a patient with an S1–S2 HV that resulted in congenital Lumbosacral junction kyphosis (CLSJK) along with a neurological bladder.

## 2 CASE PRESENTATION

A 40‐year‐old woman presented with a mild spine deformity characterized by increased lumbar lordosis (LL) since childhood, without any other symptoms. However, over the last 4 years, the patient developed gait disturbances that gradually worsened, and her trunk tilt became more pronounced during the last 2 years.

About 11 months before the surgery, the patient started experiencing urinary issues, including increased urinary residual volume, frequent urination, dribbling, and suprapubic pain. Two months later, her urinary symptoms worsened, leading to urinary retention, which necessitated frequent catheterization. In addition, the patient's gait problems and low back pain were severely affecting her daily activities.

Moreover, the patient's exacerbated gait disturbance and accompanying low back pain significantly disrupted her daily activities.

## 3 PHYSICAL EXAMINATION AND RADIOLOGICAL FINDINGS

During the physical examination, the patient exhibited a waddling gait, a low‐grade pelvic tilt, and severe LL. Notably, all lower limb forces, sensory examination, and upper motor reflexes, such as the Babinski reflex (which elicited a downward response), and clonus, were found to be normal. Additionally, the anal sphincter tone, anal wink, and deep tendon reflexes, including the quadriceps and Achilles reflexes, were also normal.

**FIGURE 1:**
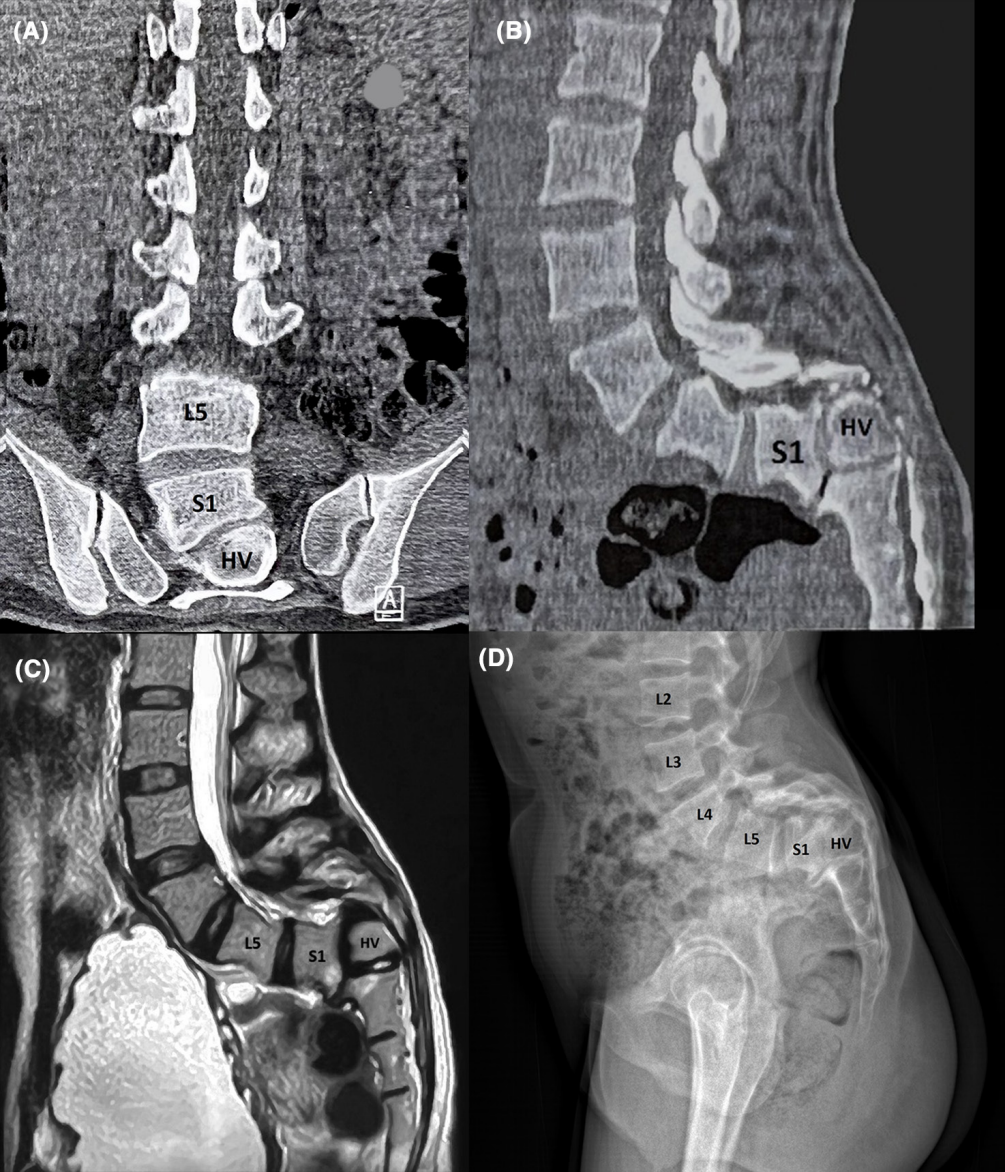
(A) Coronal view and (B) sagittal view of CT scan demonstrate the hemivertebra between S1 and S2. (C) MRI reveals the S1–S2 hemivertebra with a compressive effect on the roots and dura. (D) X‐rays show the S1–S2 hemivertebra.

**FIGURE 2:**
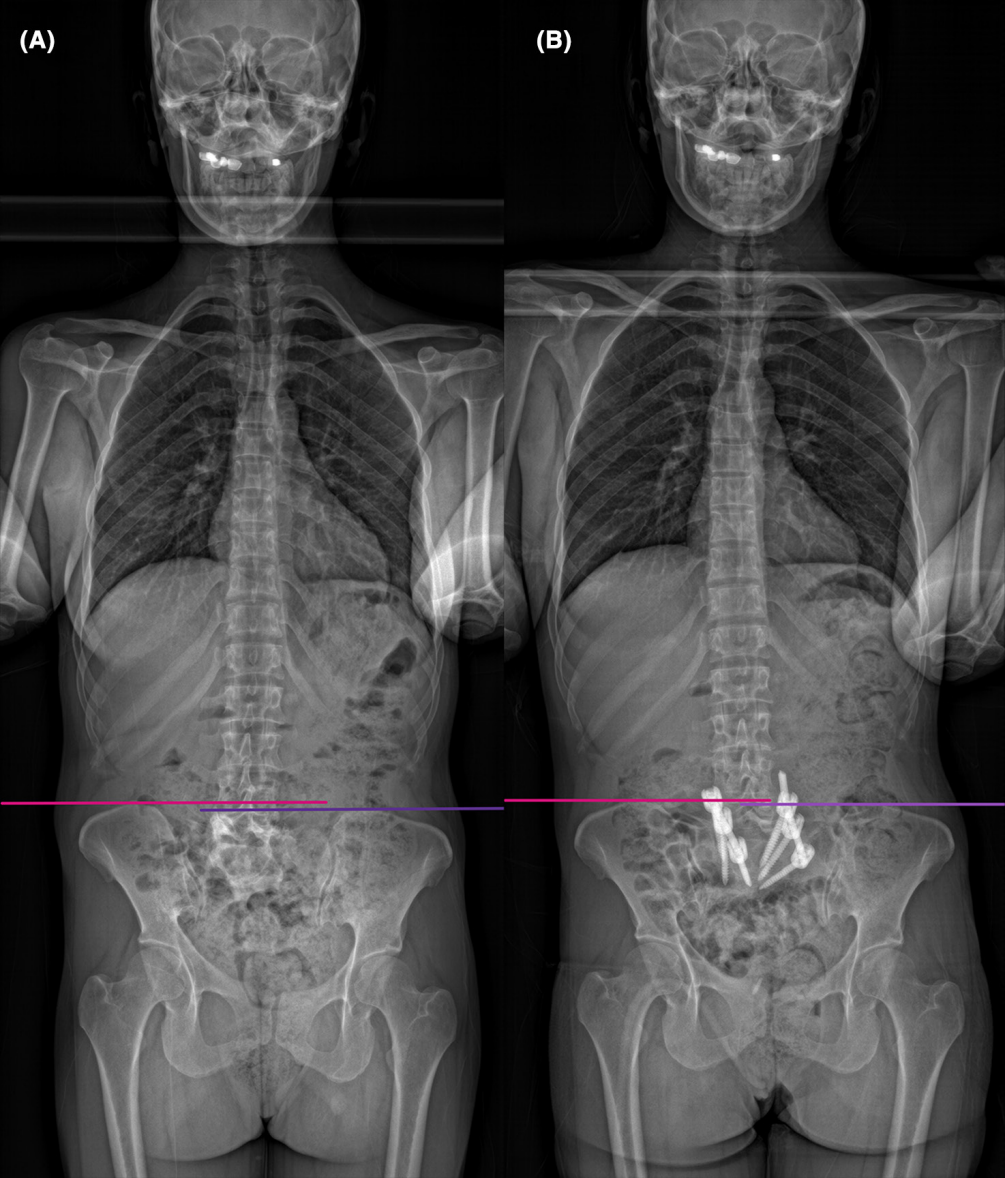
(A) The preoperative standing anteroposterior radiograph shows evidence of pelvic tilt. (B) The postoperative standing anteroposterior radiograph shows a reduction in pelvic tilt.

**FIGURE 3:**
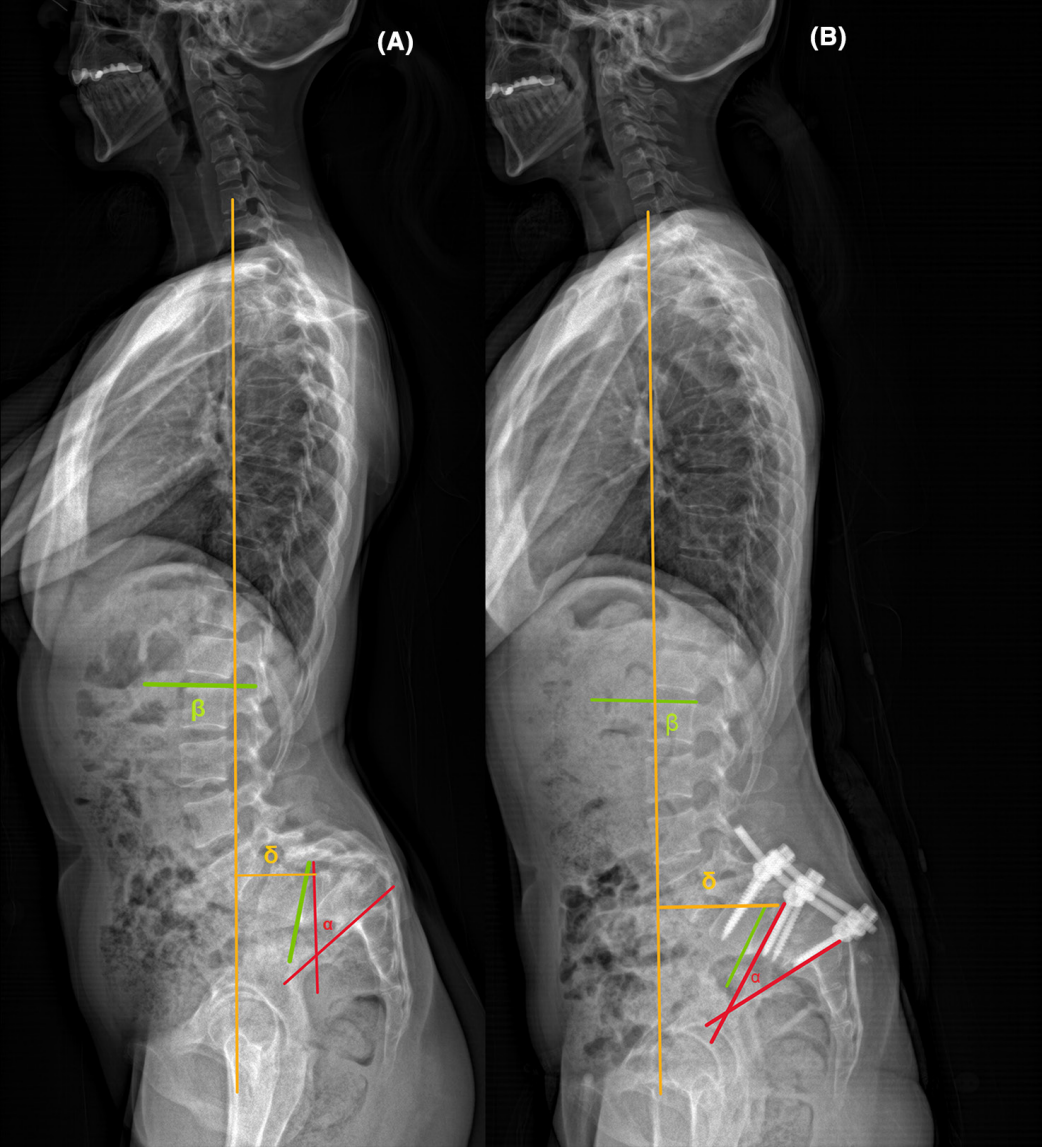
(A) Preoperative standing lateral radiograph—local kyphosis angles (LKA) (α): 44.59°, lumbar lordosis (LL) (β): 92.82°, sagittal vertical axis (SVA) (δ): 69.99 mm. (B) Postoperative standing lateral radiograph – LKA(α): 35.58°, LL(β): 67.17°, SVA (δ): 70.64 mm.

Radiological evaluation, including plain X‐ray, CT‐scan, and magnetic resonance imaging (MRI), revealed a severe CLSJK, primarily attributed to the existence of an S1–S2 hemivertebrae. (Figures 1, 2, 3).

Before the surgery, the sagittal vertical axis (SVA) was measured at 69.99 mm, and the LL and local kyphosis angles (LKA) were found to be 92.82° and 44.59°, respectively. The whole spine MRI examination before the surgery did not reveal any evidence of tethered spinal cord, Chiari malformation, diastematomyelia, or syringomyelia.

## 4 SURGICAL INTERVENTION AND POSTOPERATIVE MANAGEMENT

Given the complexity of the case and the patient's deteriorating condition, a decision was made to proceed with surgical intervention. The patient underwent surgery in a prone position, 2 g of intravenous cefazolin were administered as a prophylactic antibiotic. The level of the desired hemivertebra was determined with the C‐Arm, and a skin incision was made from L5 to S2 using the midline posterior approach. Dissection was extended down to the subcutaneous tissue and the lumbosacral fascia up to the tip of the spinous process. Self‐retaining retractors were utilized to maintain tension on soft tissues during exposure. The posterior elements were exposed subperiosteally from distal to proximal using electrocautery and periosteal elevators to detach the muscles from the posterior elements. Subsequently, the L5 transverse process and the sacral ala were exposed subperiosteally. Due to the anatomical distortion, the insertion of pedicle screws proved to be very challenging. To assist in the accurate location and orientation of the screws, multiple fluoroscopic views were used bilaterally during the preparation of the pedicle screw holes at the L5, S1, and S2 levels. Subsequently, pedicle screws were applied from L5 to S2. The ligamentum flavum was detach from the caudal and cephalad margins using small angled curets. The lamina and additional fibrocartilage around the nerve root were removed with a rongeur. The exiting L5 root was visible all the way to the lateral aspect of the foramen. The hemivertebra at the S1‐S2 level was completely resected using a rongeur and curets. The traversing S1 and S2 root were identified through the S1 and S2 foraminotomy. Partial reduction of the lumbarized S1 on S2 was achieved, aiming to address the severe LL and restore spinal alignment. Due to the excellent grip of the pedicle screws and the patient's appropriate bone quality, the insertion of an S2 alar‐iliac (S2AI) screw was not necessary.

**FIGURE 4:**
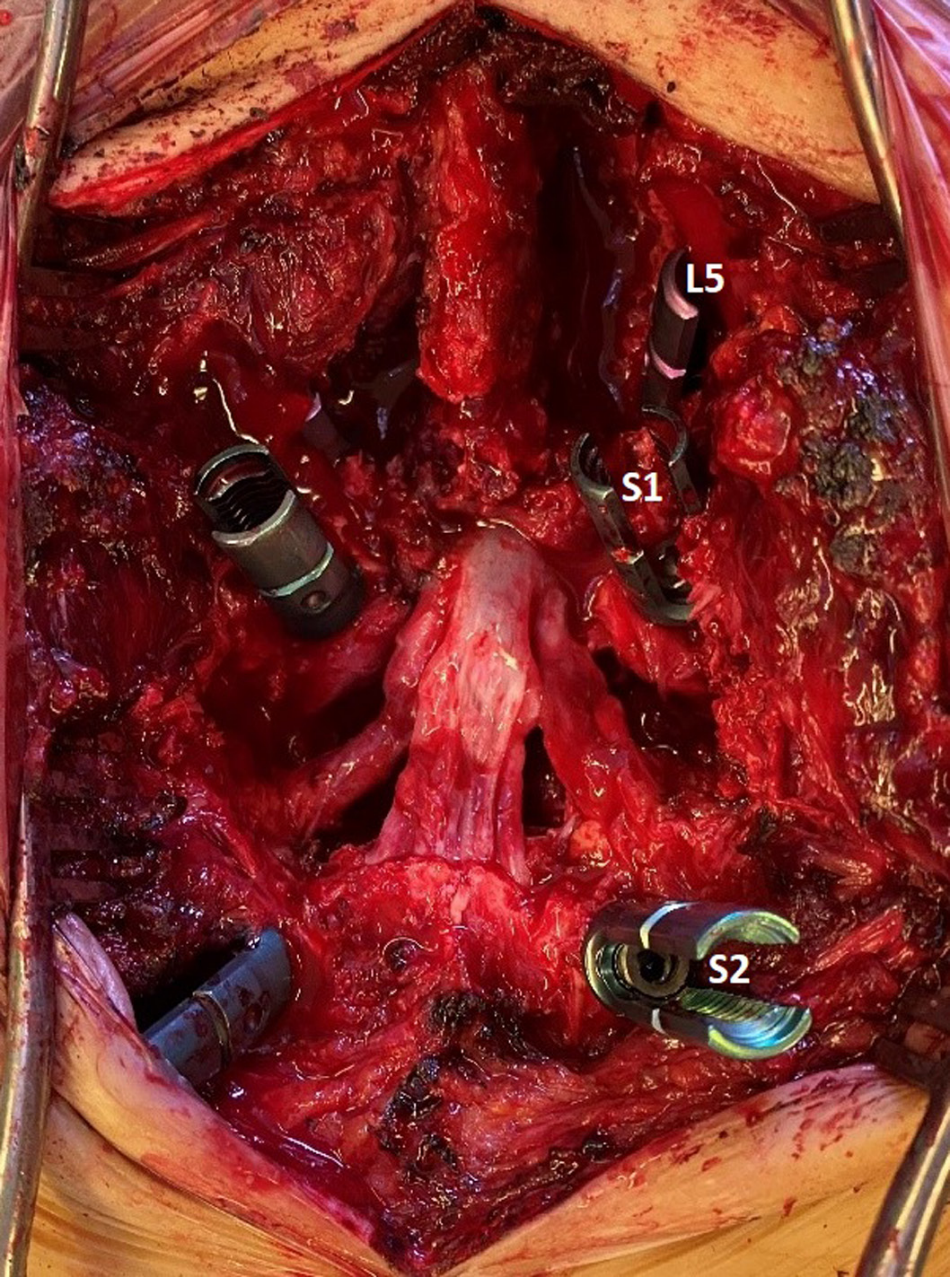
Intraoperative photograph showing the resected hemivertebra between S1 and S2, with clear visualization of the nerve roots and dura.

After completing the decompression, the transverse processes and sacral alae were decorticated, and posterior spinal fusion was performed using cancellous allograft bone chips and harvested bone from corpectomy. The graft material was soaked in an antibiotic (vancomycin) solution for at least 30 min before implantation. It was placed under direct vision in the lateral gutter (Figure 4).

Throughout the surgery, neuromonitoring control was conducted, and meticulous records were maintained until just before the surgical incision.

In the radiograph taken after surgery, SVA did not change significantly, and its value remained at 70.64 mm. Additionally, the values of LL and LKA decreased to 35.58° and 67.17°, respectively.

After the surgery, all lower limb forces, sensory examination, and upper motor reflexes, such as the Babinski reflex (which elicited a downward response), and clonus, were found to be normal. Moreover, the anal sphincter tone, anal wink, and deep tendon reflexes, including the quadriceps and Achilles reflexes, were also normal.

## 5 FOLLOW‐UP

One year of follow‐up shows significant improvement in the patient's gait, and her low back pain has been completely eliminated. Consequently, she can now perform daily activities without any restrictions. Although her urinary retention problem has not been completely resolved, there has been notable improvement compared to her condition before surgery. As a result, the patient no longer requires frequent catheterization.

## 6 DISCUSSION

In general, congenital kyphosis is a rare condition, but neurological symptoms following it are common. Sacral HV and CLSJK are extremely rare abnormalities, and their natural progression remains unclear.^12^ However, certain factors play a crucial role in determining the risk of progression, such as the location and type of hemivertebra, as well as the number of involved vertebrae. Congenital kyphosis is classified into three categories according to Winter et al.: Type I, which involves congenital failure of vertebral body formation; Type II, characterized by failure of vertebral body segmentation; and Type III, which represents a combination of both conditions.^8^, ^13^ It has been reported that approximately 25% of Type I patients have exhibited neurological symptoms. Anterior failure of vertebral body formation leads to a sharply angular kyphosis, which can be more deforming and pose a greater risk of neurological complications compared to a curve with a similar Cobb measurement. This is due to the involvement of several adjacent vertebrae resulting from an anterior failure of segmentation, producing a smoother, less obvious deformity.^8^, ^13^, ^14^ In the patient we introduced, the deformity at the dorsal side did not present clear symptoms related to HV initially. The first problematic symptom for the patient emerged as neurological symptoms in midlife.

Previously, only a limited number of cases of HV in the lumbosacral region have been reported (Table 1). Daher et al., reported a 10‐month‐old infant with anorectal malformation who underwent rectal surgery. Further examinations revealed the presence of a hemivertebra at the S1‐S2 level. However, the authors did not provide specific details about the spine problem or offer an explanation regarding the follow‐up of this condition.^6^ Karaeminogullari et al. reported a case of an 11‐month‐old boy who underwent surgical treatment for congenital heart disease and was incidentally found to have a sacral hemivertebra at the S2‐S3 level. Remarkably, there were no signs of thoracic or lumbar scoliosis. The patient was followed for 7 years, during which no symptoms such as lower back pain in either standing or seated positions were observed. Moreover, the patient exhibited normal gait, muscle power, sensation, and reflexes for the lumbar and sacral nerves.^7^ Whereas Ansari et al., reported two cases with L5‐S1 HV, both of whom presented with functional kyphosis at the lumbosacral junction. Both patients exhibited a waddling gait with flexed hips and experienced bowel and bladder difficulties, as confirmed by abnormal urodynamic testing.^15^ Interestingly, these patients displayed similar symptoms and clinical courses to our case report, with the distinction that our patient had a hemivertebra at the S1‐S2 level.

Considering the high probability of progression in the treatment of congenital kyphosis, early treatment is essential. Winter et al. described 41‐degree deformity advancement following dorsal HV in a sample of 130 patients over 6 years.^13^ Additionally, Nazareth et al. have emphasized that CLSJK, like other congenital kyphosis types, should be treated before the appearance of neurological symptoms.^9^ Therefore, early intervention is crucial to address CLSJK before neurological symptoms manifest. Although adult surgery may not completely resolve neurological problems, it can still contribute to improvement.

## 7 CONCLUSION

CLSJK following HV at the S1‐S2 level is an extremely rare condition, and no symptomatic adult patients had been reported for previously. Similar to other congenital kyphosis types, CLSJK should be treated before the onset of neurological symptoms. However, performing surgery in adulthood can still contribute to the improvement of neurological disturbances, even if complete resolution may not be achieved.

## AUTHOR CONTRIBUTIONS

**Arvin Eslami:** Data curation; investigation; writing – original draft; writing – review and editing. **Mohammadreza Chehrassan:** Methodology; project administration; supervision. **Shaya Alimoghadam:** Writing – original draft. **Mohammadreza Shakeri:** Conceptualization; methodology; project administration; supervision; writing – review and editing.

## FUNDING INFORMATION

No funds and resources were received in support of this work.

## CONFLICT OF INTEREST STATEMENT

We have no conflicts of interest to disclose.

## ETHICS STATEMENT

All procedures performed in studies involving human participants were in accordance with the ethical standards of the institutional and/or national research committee and with the 1964 Helsinki declaration and its later amendments or comparable ethical standards.

## CONSENT STATEMENT

Written informed consent was obtained from the patient to publish this report in accordance with the journal's patient consent policy.

## Data Availability

All data are included in this published article and its additional information files.

## References

[1] McMaster MJ , Ohtsuka K . The natural history of congenital scoliosis. A study of two hundred and fifty‐one patients. J Bone Joint Surg Am. 1982;64(8):1128‐1147. 7130226

[2] Faivre L , Portnoï MF , Pals G , et al. Should chromosome breakage studies be performed in patients with VACTERL association? Am J Med Genet A. 2005;137(1):55‐58. doi:10.1002/ajmg.a.30853 16015582

[3] Darouich S , Amraoui J , Amraoui N . Femoral‐facial syndrome: report of 2 fetal cases. Radiol Case Rep. 2019;14(10):1276‐1282. doi:10.1016/j.radcr.2019.08.001 31452825 PMC6704398

[4] Dentici ML , Barresi S , Niceta M , et al. Clinical spectrum of Kabuki‐like syndrome caused by HNRNPK haploinsufficiency. Clin Genet. 2018;93(2):401‐407. doi:10.1111/cge.13029 28374925

[5] Fayard C , Blondiaux E , Grigorescu R , Garel C . AIRP best cases in radiologic‐pathologic correlation: prenatal and postmortem imaging of a complex cloacal malformation. Radiographics. 2014;34(7):2056‐2063. doi:10.1148/rg.347140018 25384301

[6] Daher P , Daher R , Riachy E , Zeidan S . Do low‐type anorectal malformations have a better prognosis than the intermediate and high types? A preliminary report using the Krickenbeck score. Eur J Pediatr Surg. 2007;17(5):340‐343. doi:10.1055/s-2007-965462 17968791

[7] Karaeminoğullari O , Sahin O , Akgun RC , Karaman A , Atabey M . Sacral hemivertebra: a case report. Eur J Orthop Surg Traumatol. 2005;15:316‐318.

[8] Winter RB , Moe JH , Wang JF . Congenital kyphosis. Its natural history and treatment as observed in a study of one hundred and thirty patients. J Bone Joint Surg Am. 1973;55(2):223‐256. 4572221

[9] Nazareth A , Andras LM , Krieger MD , Skaggs DL . Bilateral congenital posterior hemivertebrae and lumbar spinal stenosis treated with posterior spinal fusion and instrumentation. J Am Acad Orthop Surg Glob Res Rev. 2019;3(10):e19.00054. doi:10.5435/JAAOSGlobal-D-19-00054 PMC6855501 31773076

[10] Bollini G , Docquier PL , Viehweger E , Launay F , Jouve JL . Lumbosacral hemivertebrae resection by combined approach: medium‐ and long‐term follow‐up. Spine (Phila Pa 1976). 2006;31(11):1232‐1239. doi:10.1097/01.brs.0000217616.17692.a0 16688037

[11] Acharya S , Palukuri N , Gupta P , Kohli M . Transcranial motor evoked potentials during spinal deformity corrections‐safety, efficacy, limitations, and the role of a checklist. Front Surg. 2017;4:8. doi:10.3389/fsurg.2017.00008 28243591 PMC5303707

[12] Goldstein I , Makhoul IR , Weissman A , Drugan A . Hemivertebra: prenatal diagnosis, incidence and characteristics. Fetal Diagn Ther. 2005;20(2):121‐126. doi:10.1159/000082435 15692206

[13] Winter RB . Congenital kyphosis. Clin Orthop Relat Res. 1977;128:26‐32. 598164

[14] McMaster MJ , Singh H . Natural history of congenital kyphosis and kyphoscoliosis. A study of one hundred and twelve patients. J Bone Joint Surg Am. 1999;81(10):1367‐1383. doi:10.2106/00004623-199910000-00002 10535587

[15] Ansari SF , Rodgers RB , Fulkerson DH . Dorsal midline hemivertebra at the lumbosacral junction: report of 2 cases. J Neurosurg Spine. 2015;22(1):84‐89. doi:10.3171/2014.9.Spine1411 25343409

